# A collaborative care psychosocial intervention to improve late life depression in socioeconomically deprived areas of Guarulhos, Brazil: the PROACTIVE cluster randomised controlled trial protocol

**DOI:** 10.1186/s13063-020-04826-w

**Published:** 2020-11-05

**Authors:** Marcia Scazufca, Carina Akemi Nakamura, Tim J. Peters, Maiara Garcia Henrique, Antônio Seabra, Ehidee Gomez La Rotta, Renato M. Franzin, Daniele Ferreira Martins, Pepijn Van de Ven, William Hollingworth, Ricardo Araya

**Affiliations:** 1 Hospital das Clinicas HCFMUSP, Faculdade de Medicina, Universidade de Sao Paulo, Sao Paulo, Brazil; 2 Faculdade de Medicina FMUSP, Universidade de Sao Paulo, Sao Paulo, Brazil; 3 University of Bristol Medical School, Bristol, England; 4 Departamento de Engenharia Eletrica, Escola Politecnica, Universidade de Sao Paulo, Sao Paulo, Brazil; 5 Faculty of Science and Engeneering, University of Limerick, Limerick, England; 6 Institute of Psychiatry Psychology and Neurosciences, King’s College London, London, England

**Keywords:** Depression, Collaborative care, Elderly, Randomised controlled trial, Primary healthcare, Protocol, LMIC

## Abstract

**Background:**

The elderly population has been growing in most low- and middle-income countries (LMIC), and depression is a common condition among these populations. The lack of integration between mental health and primary healthcare services and the shortage of mental health specialists in the public health system contribute to underdiagnosis and undertreatment of depression. One of the strategies to reduce this gap is task shifting and collaborative care treatments. This study therefore aims to evaluate the effectiveness and cost-effectiveness of a collaborative care psychosocial intervention to improve the clinical management of depression among elderly people in poor neighbourhoods in Guarulhos, Brazil.

**Methods:**

Two-arm, cluster randomised controlled trial with Basic Health Units as the clusters and a 1:1 allocation ratio. Twenty Basic Health Units have been randomly selected and randomised to control or intervention arms. We aim to recruit 1440 adults (72 per cluster) aged 60 years or over identified with depression (9-item Patient Health Questionnaire (PHQ-9) score ≥ 10). The control arm participants will receive an enhanced usual care, while the intervention arm participants will receive an enhanced usual care and a 17-week psychosocial intervention programme delivered at home by community health workers with the help of an application installed on tablet computers. The primary outcome is the proportion with depression recovery (PHQ-9 < 10) at 8 months’ follow-up. We will also assess the maintenance of any earlier clinical gains and the cost-effectiveness of the intervention at 12 months.

**Discussion:**

This is the first randomised trial to investigate a collaborative care intervention to treat depression among poor elderly in LMIC/Latin America. This is a major public health problem worldwide, but in these countries, there are no locally tested, evidence-based interventions available to date.

**Trial registration:**

International Standard Randomised Controlled Trial Number ISRCTN57805470. Registered on 25 April 2019

## Administrative information

The order of the items has been modified to group similar items (see http://www.equator-network.org/reporting-guidelines/spirit-2013-statement-defining-standard-protocol-items-for-clinical-trials/).

**Table:** 

Title {1}	A collaborative care psychosocial intervention to improve late life depression in socioeconomically deprived areas of Guarulhos, Brazil: the PROACTIVE cluster randomised controlled trial protocol.
Trial registration {2a and 2b}.	International Standard Randomised Controlled Trial Number (ISRCTN57805470).
Protocol version {3}	Version 2.0, 16th March 2020.
Funding {4}	São Paulo Research Foundation (FAPESP, process number 17/50094-2) Medical Research Council (MRC, process number MR/R006229/1).
Author details {5a}	(1) USP Medical School, University of São Paulo, Brazil (2) Engineering School, University of São Paulo, Brazil (3) Faculty of Science and Engineering, University of Limerick, Ireland (4) Bristol Medical School, University of Bristol, United Kingdom (5) Institute of Psychiatry, Psychology and Neuroscience, King’s College London, United Kingdom
Name and contact information for the trial sponsor {5b}	Sponsors: University of São Paulo (USP), Brazil King’s College London (KCL), United Kingdom Principal Investigators contact: Dr Marcia Scazufca, University of São Paulo, Brazil scazufca@usp.br Professor Ricardo Araya, King’s College London, United Kingdom ricardo.araya@kcl.ac.uk
Role of sponsor {5c}	The sponsors and funders have no roles in the design, conduction, analysis and interpretation, or in the writing or decision to submit the manuscript for publication.

## Introduction

### Background and rationale {6a}

The rapid growth of the elderly population is a reality in most low- and middle-income countries (LMIC). Brazil had more than 30 million people aged over 60 years in 2017 (14% of the population, with 8% being women) [1, 2].

Depression is the leading cause of disability worldwide [3] and among older adults is a common chronic condition with a prevalence around 8% according to the World Health Organization (WHO) [4]. A National Health Survey-Brazil (2013) found a higher prevalence of depression diagnosis in people aged between 60 and 64 years (11.1%) and 65 and 74 years (9.9%) compared with the other age groups over 18 years (3.9–8.8%) [5, 6]. Moreover, a study with older adults living in poor neighbourhoods in São Paulo, Brazil, found that 5% of this population met the criteria for a depressive episode and that identification of depression symptoms was low, with less than 5% of those who met these criteria being diagnosed by primary care services [7].

The existing primary care model in Brazil, the Family Health Strategy (FHS) framework, puts emphasis on regular household visits to encourage prevention with the central role played by community health workers (CHWs), early identification of health problems and improved adherence to ongoing treatments. However, mental health care is still not adequately integrated into the primary care services, and there is a scarcity of trained mental health resources working in the public system [8]. Thus, depression in later life is often unidentified and left untreated despite the vast array of adverse social, economic and health consequences [7, 9, 10].

One strategy proposed by the World Health Organization in order to reduce this treatment gap and alleviate shortages of mental health specialists is task shifting from physicians to nurses or other non-medical providers such as CHWs [11]. In the context of mental health care, task shifting can reduce the workload on psychiatrists and clinical psychologists and increase the number of people receiving mental health interventions delivered by trained non-specialists with appropriate supervision [12, 13].

The most effective primary care treatments for depression have used collaborative and stepped-care models developed in high-income countries [14–17]. Given the socio-cultural and healthcare system differences, it is not appropriate to simply generalise from evidence gathered from these studies. Thus, it is imperative that local cost-effective solutions are developed to address depression in LMIC.

To date, several collaborative task-shifted programmes have been tested in LMIC with good results, but none of these studies was conducted with older adults [18–21]. For this reason, we conducted a pilot study to investigate the acceptability and feasibility of a psychosocial collaborative care intervention to treat depression among elderly people attending Unidades Básica de Saúde (UBS) or Basic Health Units in São Paulo, Brazil [22, 23]. Based on the positive results of this pilot study, we are proposing to conduct what we believe will be the first task shifting, stepped-care and collaborative care intervention aimed at treating late-life depression in LMIC/Latin America.

## Objectives {7}

This study aims to evaluate the effectiveness and cost-effectiveness of a psychosocial intervention to improve the clinical management of depression among elderly people in poor neighbourhoods in Guarulhos, Brazil.

## Trial design {8}

It is a parallel two-arm, cluster, superiority randomised controlled trial with Basic Health Units as the clusters and a 1:1 allocation ratio.

## Methods: participants, interventions and outcomes

### Study setting {9}

The trial will be conducted in the city of Guarulhos, situated in the metropolitan region of São Paulo city, Brazil. The estimated population of Guarulhos in 2019 was 1.38 million, of whom 8.2% are aged over 60 years [24]. Around 39% of the population of Guarulhos is covered by total or partial private health insurance, not totally dependent on the public health system [25].

There is a total of 69 UBSs across the city of Guarulhos [26]. Of those 69 UBSs, 47 work as a Family Health UBS that covers about 32% of the population [27]. Half of them (24 UBSs) [26] have four or more Family Health Teams (FHTs), which are multi-professional teams providing comprehensive primary care to the population who live in the catchment areas. The other 22 UBSs follow the traditional Brazilian primary healthcare model, where most of the care is provided through appointments with physicians at the UBS and the outreach activities are far less prominent [28].

### Eligibility criteria {10}

#### Clusters

UBSs with professionals working exclusively on the FHS model with at least four FHTs embedded within each one.

#### Individuals who will perform the intervention

The CHW is a lay health professional who is part of the FHT. CHWs often live in the same territory covered by the UBS. Their main roles involve mapping a defined area of the FHT where around 750 families live (called micro-area), visiting these households monthly and giving advice on health promotion and disease prevention for all residents of the households covered by their micro-area [29]. All CHWs who are working in the selected FHTs are eligible to participate in this study. The UBS managers and FHT members will be responsible for selecting three CHWs per FHT to conduct the home psychosocial intervention.

#### Participants

Inclusion criteria:

1. Individuals 60 years or older registered with the selected FHTs were eligible for screening
2. Individuals with a score of 10 or above, a known threshold for depression, on screening with the 9-item Patient Health Questionnaire (PHQ-9) [30]

Exclusion criteria:

1. Individuals whose partner (or another person who lives in the same household) has already been included in the study
2. Individuals identified with acute suicidal risk
3. Individuals with hearing or vision loss
4. Individuals unable to communicate (non-native speaker, cognitive impairment or psychotic symptoms)
5. Individuals unable to engage in the trial for the period of 12 months (terminal illness, partner with terminal illness, plans to move out to another area or full-time working)

### Who will take informed consent? {26a}

All individuals aged 60 years or older will be asked to consent to participate and be audio recorded by the research assistants (RAs) before both screening and baseline assessments. If the screening interview is conducted by telephone, only the verbal consent will be recorded. When the screening is conducted face-to-face, participants will be asked to sign an informed consent form. Moreover, a new informed consent to enter in the trial will be obtained at the baseline assessment.

### Additional consent provisions for collection and use of participant data and biological specimens {26b}

On the consent form, participants will be asked if they agree to the use of their data should they choose to withdraw from the trial. Participants will also be asked for permission for the research team to share relevant data with people from the Universities taking part in the research or from regulatory authorities, where relevant. This trial does not involve collecting biological specimens for storage.

## Interventions

### Explanation for the choice of comparators {6b}

The control arm participants will receive an enhanced usual care, while the intervention arm participants will receive both an enhanced usual care and a collaborative care psychosocial intervention. The main component of the intervention is a psychosocial programme delivered at home by trained CHWs who have additional support through weekly supervision and an application installed on tablet computers.

### Intervention description {11a}

#### Enhanced usual care

This comprises improved recognition of depression by the research team (screening with the PHQ-9 to identify depression) followed by usual care. The research team will send a list with the names of the individuals included in the study to UBS managers (in the control and intervention arms). The research team will not interfere with usual care—for instance, prescription of medications, CHW usual monthly home visits or access to family doctors or mental health specialists.

#### Psychosocial intervention

The psychosocial intervention was designed to provide care for depressed older adults registered with UBSs within the FHS framework in Brazil. The intervention is planned to last 17 weeks. Core principles of the intervention are:

- Task shifting: the CHWs are the bond between the FHT members and the community and are involved in the care of chronic conditions. As they visit homes routinely, they have been chosen to deliver the psychosocial intervention
- Collaborative care: the CHWs will work collaboratively with all other members from their FHTs to deliver the intervention
- Stepped care: the intensity of care management is tailored according to needs. Those who need more receive more and vice versa. The FHT will decide if the participant needs further support. The participant will continue receiving the psychosocial intervention in situations where he/she is referred to see the family doctor or mental health specialist

The intervention will be delivered at home for several reasons: older adults have difficulties travelling; an intervention at home is likely to improve adherence; the CHW visits homes regularly; and it provides an opportunity to contact caregivers, if available.

The intervention is also based on:

- Measurement-based care—depression symptoms are measured in all home sessions with the PHQ-9 instrument
- Psychoeducation—education about depression, relapse prevention strategies and simple ways to cope with depression symptoms and associated problems
- Behavioural activation technique—educate about the importance of engaging in pleasant or meaningful activities, thus increasing positive interactions with their environment
- Use of technology—the CHWs will deliver the intervention with the support of an application installed on tablets [22, 23]

The intervention is divided into initial (3 weeks) and second (14 weeks) phases. The initial phase includes three, weekly home meetings and is the same for all participants. The goal of the programme’s initial phase is to start psychoeducation—that is, what are the main symptoms of depression, how participants can deal with their problems associated with depression, and develop simple strategies to cope with these problems. When the initial phase is completed, the participant will have access to either low- or high-intensity second phase regimens. If the participant shows improvement during the initial phase (PHQ-9 < 10 in both sessions 2 and 3), the low-intensity treatment regimen will be offered with five additional meetings (three every other week followed by two monthly). If no improvement is shown (PHQ-9 ≥ 10 in session 2 and/or session 3), the participant will proceed to the high-intensity regimen having eight additional meetings (six weekly followed by two monthly). Thus, regardless of the regimen, the intervention will last around 17 weeks in total.

During the second phase, the participants will learn about behavioural activation and relapse prevention techniques, that is, how participants can identify that symptoms are returning. The choice of behavioural activation was due to the demonstrated feasibility and efficacy of this technique for the treatment of depression [31, 32] and its acceptability by participants and health workers in our pilot study [22]. It is a simple technique to apply and requires only a short period of professional training [12, 33].

Multimedia material (24 short videos with animations) were developed to be used as support material and to be watched during the sessions. The contents are introduced by an anchor and illustrated by animations. Language was adapted to be easily understood by participants. To assist with potential vision problems, we will be using tablets with a minimum screen size of 9 in. (about 23 cm).

Before the intervention starts in each UBS, the 12 CHWs participating in the study will receive training to conduct the intervention. Training will be conducted at the UBS by a clinical supervisor and an information technology specialist, during three group sessions of 6 h each. During the period of the intervention, the CHWs will also receive group supervision at the UBS by a mental health specialist. The maximum number of CHWs in each group supervision will be six. Initially, the supervision will be weekly, and then fortnightly or less frequently depending on the need of CHWs. A standard operating procedure (SOP) was developed for planning, managing and conducting the intervention.

### Criteria for discontinuing or modifying allocated interventions {11b}

#### Cluster level

At UBS or FHT request.

#### Individuals who will perform the intervention

At CHWs or UBS manager request.

#### Individual participants

Moving out of the catchment area of the UBS during the intervention or participant request. In situations where the participant moves to a neighbourhood close to the UBS and can attend the remaining sessions at the UBS, the intervention will not be discontinued.

### Strategies to improve adherence to interventions {11c}

Participants will be attributed to the CHWs according to the geographical area where each CHW works, but whenever possible, the CHWs will also be able to choose the participants they already know or they feel more comfortable with. The application is interactive; during each session, participants can choose from pre-defined options of depression-related discussion topics. Thus, we will be able to provide a more enjoyable experience for all people involved, while at the same time retaining a pragmatic approach since this is very likely how such an intervention would be applied if adopted in the future. The date and time of each appointment will be agreed between the participant and the CHW. The home-based intervention prevents the displacement of the participants and minimises barriers to access. The support of an application to watch short videos will be important for the understanding of the intervention’s content. Participants will also receive a booklet with written information about the intervention that can be used to write down the planned activities to be performed between the sessions and the next appointments with the CHW.

### Relevant concomitant care permitted or prohibited during the trial {11d}

Usual care delivered by the FHT and any other form of health care received by the participant will continue without any restrictions in both study arms, including the prescription of antidepressant drugs and scheduling of medical appointments.

### Provisions for post-trial care {30}

Participants will continue to receive care from the FHTs after the trial termination.

### Outcomes {12}

#### Primary outcome

The primary outcome is the proportion with depression recovery (PHQ-9 < 10) measured 8 months after the inclusion in the study.

#### Secondary outcomes

The proportion with depression recovery (PHQ-9 < 10) at 12 months will assess the maintenance of any earlier gains. The following additional secondary measures will be completed at 8 and 12 months and compared between the two arms: European Quality of Life five-dimensional questionnaire, Five-level version (EQ-5D-5L) [34]; ICEpop CAPability measure for Older people (ICECAP-O) [35]; Behavioural Activation for Depression Scale—Short Form (BADS-SF) [36]; Generalized Anxiety Disorder-7 (GAD-7) [37]; and stressful life events [38]. We will assess the cost-effectiveness of the intervention at 12 months.

### Participant timeline {13}

**Fig. 1:**
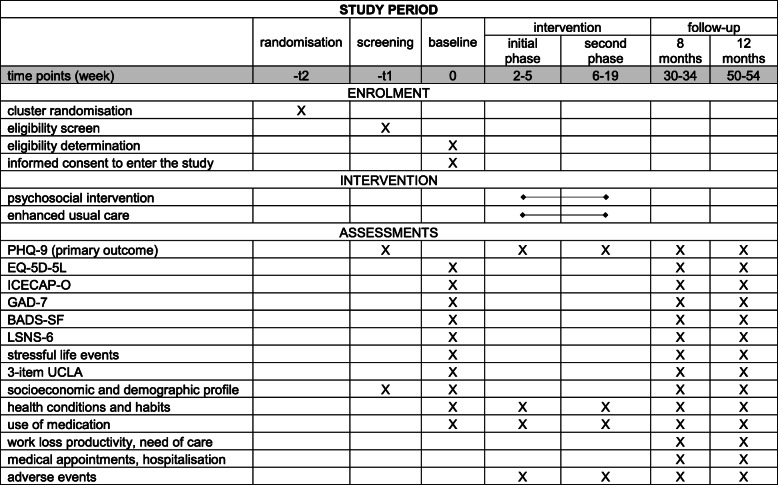
Schedule of enrolment, interventions and assessments. PHQ-9, 9-item Patient Health Questionnaire; EQ-5D-5L, European Quality of Life five-dimensional questionnaire, five-level version; ICECAP-O, ICEpop CAPability measure for Older people; GAD-7, Generalized Anxiety Disorder-7; BADS-SF, Behavioural Activation for Depression Scale—Short Form; LSNS-6, Lubben Social Network Scale-6—LSNS-6; 3-item UCLA, 3-item University of California, Los Angeles (UCLA) loneliness scale

Participants will have the baseline interview no more than 28 days after the screening. In the intervention arm, participants will have their first intervention session about 2 weeks after their baseline interview. Follow-up data collection is scheduled to occur at two time points, 8 and 12 months after consenting into the trial, for both intervention and control arms. The 8-month follow-up will be conducted between 30 and 34 weeks, and the 12-month follow-up between 50 and 54 weeks. The total length of participant commitment is therefore up to 54 weeks. The timeline of the study and assessments are given in Fig. 1.

### Sample size {14}

A total sample size of 374 individuals would detect a 15 percentage point difference (25% versus 40%) in recovery between the control and intervention arms at 8 months, with 85% power and 2-sided 5% significance level. A 15 percentage point difference in recovery rates is considered clinically meaningful. It is also achievable given the results of previous similar studies including our pilot [14, 15, 18, 22, 39, 40]. We anticipate 15% attrition, which is readily achievable according to the pilot study, which yields a corrected total sample size of 440. We plan to include four FHTs in each UBS, and therefore, the UBSs may need to be selected applying a probability proportionate to size procedure [41] depending on the variation in size of the eligible UBSs (though in the event we have needed to include all 24 UBSs that met the inclusion criteria). All of the following estimates are based on our primary care survey [42] and our pilot study in São Paulo [22]. We expect that on average 10% of the individuals registered with each UBS will be aged 60 years or older and the prevalence of older adults scoring above nine on the PHQ-9 is 10% in this population. On average, there are approximately 400 individuals in the eligible age range and therefore 40 individuals potentially eligible in each FHT, though this is likely to reduce to 24 once other entry criteria are applied. Based on pilot data, of these 24 eligible individuals, we expect that at least 20 will consent to enter the trial. However, experience in the pilot indicates that each CHW is likely to be able to manage a maximum of six participants at any given time in the trial, which means we will only be able to recruit 18 of these 24. Based on our previous study [42] and the literature, an intra-cluster correlation coefficient of 0.03 is reasonable for the binary PHQ-9 recovery outcome [43]. With four FHTs per UBS, this leads to a cluster size of 72 with 18 participants from each FHT; a design effect of 3.13 is therefore indicated. This leads to a total inflated sample size of 1378 individuals, which would require 19.14 clusters. With 20 clusters (10 in each arm), we anticipate a total of 1440 participants, yielding a power of 86.5% for our target difference; equivalently, we would retain 85% power if the attrition rose to 19%. Moreover, if the attrition in the pilot (7%) were to be repeated in the main trial, we would have a power of approximately 90% to detect the target difference of 25% versus 40%.

### Recruitment {15}

#### Clusters

Twenty UBSs (clusters) will participate in the study and four UBS will be (randomly) selected as reserves. Within each one of the 24 UBSs, four FHTs will be randomly sampled. Thus, a total of 80 FHTs will participate in the study.

#### Individuals who will perform the intervention

Within each of the sampled FHTs, selection of the CHWs to be involved in the study (three per FHT and hence 12 per UBS) will be decided by the UBS manager and the FHT members, consistent with what would happen in practice. A total of 120 CHWs will be selected to conduct the intervention. Additional CHWs will be trained to replace the selected CHWs for a short time in case of annual leave, health issues or any other situation where replacement is required. There is no need to select CHWs in the control arm.

#### Participants

The Health Secretariat of Guarulhos provided to the study investigators a list with names and contact information of the individuals aged 59 years or older registered with all FHT randomly selected to participate in the study. The list, generated by the electronic health system database (called e-SUS), will be entered into the PROACTIVE database in a random order, to avoid conducting the recruitment using the original sequence of the names contained on the e-SUS. Recruitment of the participants from this list of individuals from both arms will be conducted in two phases (screening and baseline assessments) and in two waves (in order not to over-burden CHWs). Each wave will last for a period of 30 weeks, comprising five blocks of 6 weeks. During each block, participants of two intervention and two control UBSs will be recruited; the second wave will be a repeat of the first. Participants in each wave will only be contacted if they are 60 years old or over on the date of recruitment. In each wave, we will recruit 720 participants (360 in each study arm, a total of 36 participants per UBS, and nine participants per FHT).

From the pilot study [22], we recruited 6% of the total population of those at least 60 years of age. To achieve our targets for this study, we need to assess 24,000 individuals, and a sub-contracted company will conduct the screening, baseline and follow-up assessments according to an agreed and detailed protocol and contract. We decided to sub-contract as the number of people who will be screened for depression is large and exceeds the capacities of our team. A SOP for data collection during the interviews has been developed to help the RAs.

##### Screening assessment

As information from e-SUS database is not frequently updated, we will ask the UBS managers and CHWs to pre-screen a list of around 150 names per FHT in order to identify individuals who we will not be able to find (dead or moved to another area) and those who present clinical exclusion criteria. We will also ask the CHWs to update the individuals’ contact information.

The RA from the sub-contracted company will follow the random order of names on the list to contact individuals for screening. The contact will be by phone or home visit, and there will be at least one contact attempt through a home visit before excluding any individual from the study.

##### Baseline assessment

If the individual scores 10 or above on the PHQ-9 (screening interview) and does not present any exclusion criteria, he/she will be invited to the baseline assessment. All baseline interviews will be conducted by a home visit. If the screening interview is conducted by phone, at the end of the interview, the RA will schedule a home visit to conduct the baseline assessment. If the screening is conducted by a home visit, the researcher will proceed to the baseline assessment on the same day of the screening whenever possible, after obtaining the participant’s written consent to enter the trial. In situations where the individual prefers to continue with the baseline interview on another day, the RA will schedule a new home visit.

Soon after recruitment is completed in each UBS, the study coordinator will send a list to UBS managers (both study arms) with the names of all individuals from their clinics who agreed to participate in the study. The UBS manager will be advised by the study coordinator to inform the relevant FHT members about the individuals under their care who are participating in the study. From this point, the FHTs will manage these individuals with depression according to usual practice. In the intervention arm, besides informing UBS managers about the participants included in the study, the clinical supervisor from each UBS will also receive a list with the names of all study participants before assigning them to the CHW who will conduct the psychosocial intervention.

## Assignment of interventions: allocation

### Sequence generation {16a}

#### Clusters

UBSs will be identified, approached to participate and then randomised at the beginning of the study. For stratification, we will use the educational level (percentage of individuals with no education or who had completed a literacy programme for adults) collected through the e-SUS database of the individuals 60 years or older registered with each UBS. Stratification will be into two sets of UBSs (according to whether they are above or below the median on this educational variable). Four UBSs will be randomly selected as reserves (two from each stratum), in case a UBS decides subsequently to not take part in the study. Of those 20 remaining UBSs, 10 will be randomly allocated to each arm of the trial (five from each stratum). Also, four FHTs within each UBS will be randomly selected to be part of the study.

### Concealment mechanism {16b}

Not applicable at the level of the individual participant given the nature of this cluster randomised trial, though the UBSs (clusters) will all be recruited before allocation to the trial arm is conducted.

### Implementation {16c}

The Bristol Randomised Trials Collaboration (through a merger, now part of the Bristol Trials Centre), which is a UK Clinical Research Collaboration fully registered Clinical Trials Unit, will conduct the randomisation of the UBSs and FHTs, by an individual (TJP) not involved in the trial recruitment process at any stage. Allocation/selection will therefore be conducted remotely by an individual not involved in the recruitment and will be conducted only after all UBSs have formally confirmed their participation in the trial.

## Assignment of interventions: blinding

### Who will be blinded {17a}

Complete blinding of participants or CHWs to trial allocation is impossible given the nature of this intervention. To decrease the risk of observer bias at follow-up, the two follow-up assessments will be made by RAs who are not involved in the initial recruitment or in delivering the intervention. We will rotate these RAs so that unless it is unavoidable the same researcher does not conduct any interview with the same participant more than once. We will also ensure that the primary data analysis is completed blind to trial allocation.

### Procedure for unblinding if needed {17b}

Unblinding will occur only after the analysis of primary endpoints has been completed, and likewise after as many of the secondary outcomes are considered as is feasible before unblinding becomes impossible to avoid.

## Data collection and management

### Plans for assessment and collection of outcomes {18a}

#### Collection of trial data

Individual data will be gathered and stored electronically in an information technology system developed for the management of this study. Participants consenting to the trial will be informed of this and receive an ‘ID number’ when entering the study. All data will be linked using ID codes to identify the person. A list of IDs will be kept in a secure place with access only to the joint principal investigators. Data will be captured via digital recorders, tablets and on paper when necessary. Screening, baseline and follow-up data will be collected by a sub-contracted company using a software provided by the research team, and all data will be stored in the study server. The sub-contract with the company will ensure that access and protection of the data is consistent with Medical Research Council guidelines and National Health Council (Conselho Nacional de Saúde—Brazil) (Resolution 196/96-2012) regulations. A quality control assessment of a sample of the audio recordings will be conducted by an independent researcher trained by the research team. The main aim of this quality control is to ensure that all procedures have been correctly followed by the RAs and all information has been given to the participants. The research team will also supervise the work of this company using a web interface developed to follow live updates of the assessment’s status.

All data will be kept in a secured server at the University of São Paulo. Digital files will be stored anonymised in password-protected directories on secure computers.

We will use data recorded routinely with the app (number and duration of psychosocial treatment sessions, other actions performed at the sessions) to assess the fidelity with which the intervention was delivered. A detailed record of all clinical data will be kept. We will record the training time for all staff involved in the study.

#### Instruments

The three main instruments used in this study are the PHQ-9 (at the screening and follow-up assessments), the EQ-5D-5L and the ICECAP-O.

The PHQ-9 [30, 44] is a well-validated brief depression measure extensively used in clinical research, including Brazil [45], among other reasons because it is sensitive to changes over time [44, 46]. Acute suicide risk will be assessed with a standardised questionnaire whenever the ninth question of the PHQ-9 is scored 1 or more.

The EQ-5D [34, 47] is a generic preference-based outcome measure that provides a common metric for policy-makers to compare the cost-effectiveness of treatment in diverse clinical settings. The EQ-5D is sensitive to changes in mental health outcomes in the literature [48, 49], and the new five-level version was correlated with changes in the PHQ-9 scores in the pilot study [22].

The ICECAP-O [35] measure will be used to assess broader wellbeing/capability (for example, independence, security) of participants. We have registered the intention to use both EQ-5D and ICECAP-O instruments with the developers of these instruments.

We will also collect information on participants’ sociodemographic profile, health condition, anxiety symptoms (GAD-7) [37], social network (Lubben Social Network Scale-6—LSNS-6 [50, 51]), stressful life events [38], smoking habits, alcohol use (Alcohol Use Disorders Identification Test-Consumption—AUDIT-C) [52], disability, behaviour activation (BADS-SF) [36], loneliness (3-item University of California, Los Angeles (UCLA) loneliness scale) [53], work loss productivity, use of private care, need of care, use of medication for chronic conditions, consultations with healthcare professionals and admission to hospital. The economic assessment will also include resources used during the CHW training and supervisory sessions and data collected from medical records and the public health system databases (consultations and procedures).

### Plans to promote participant retention and complete follow-up {18b}

We will inform participants from both arms about the approximate date of the two follow-up interviews. Follow-up assessments will ideally be conducted face-to-face, but it can also be conducted by phone when necessary. We decided to include a time ‘window’ to reflect the logistical realities of the context and to maximise response to follow-up. If any participant from either arm is not contactable for the follow-up interview, we will contact the neighbours and the UBS managers to confirm current contact details.

### Data management {19}

The electronic data will be stored in our server, which has several mechanisms to ensure that data remain confidential and inaccessible to others than those with access rights. The principal investigators will be responsible for data security. All computers used in this project will be password protected, and access to data files archived will also be password protected. There will be a database backup updated daily and stored in a different secure server at the University of São Paulo and mirrored on a server in Limerick, Ireland, where the information technology platform has been developed. The study will be compliant with the UK’s and Brazil’s regulations on research data management. A summary of the data management will be included in the Informed Consent Form given to participants.

We will create a data dictionary, with detailed information on labelling and codes, and describe data, annotations and variables in such a way as to make possible to use by authorised third parties.

Once data are entered to the main database, it will be locked and no further changes are allowed to the original version, albeit with (auditable) changes made in subsequent versions following (usual) data checking processes.

### Confidentiality {27}

All data storage in Brazil will be compliant with the National Commission of Ethics in Research (Comissão Nacional de Ética em Pesquisa), Resolution 466/2012 security policies. All data kept in computers will be anonymised. Paper records will be kept to a minimum and stored in secure storage facilities. Any personally identifiable paper record will be stored separately from anonymised paper records. All electronic records will be stored on password-protected servers. All data will be encrypted to ensure its security. We will put in place any required limitations on data sharing that serve to safeguard the privacy of research participants.

The University of São Paulo has a modern network infrastructure and clear procedures to ensure confidentiality of the data. Parallel storage systems are located at a different location and backed up regularly. Access to data files is controlled by login and password authentication. The data generated and collected through this project will be maintained with the level of security available.

### Plans for collection, laboratory evaluation and storage of biological specimens for genetic or molecular analysis in this trial/future use {33}

There will be no biological specimen collection for this clinical trial.

## Statistical methods

### Statistical methods for primary and secondary outcomes {20a}

#### Primary analysis

The analysis and presentation of data will be in accordance with the Consolidated Standards of Reporting Trials guidelines [54], with the primary comparative analysis being conducted on an intention-to-treat basis (including only those individuals with known primary outcome and emphasis placed on confidence intervals for between-arm comparisons). Descriptive statistics of demographic and clinical measures will be used to ascertain any marked imbalance between the trial arms at baseline. The primary comparative analysis will employ random-effects multivariable logistic regression on the primary outcome to obtain an odds ratio of recovery between the arms at 8 months after adjusting for baseline PHQ-9 scores, stratification and cluster design.

#### Secondary analyses

Other secondary analyses will include (a) comparison of the primary outcome measure at 12 months and using the PHQ-9 score as a continuous variable at both follow-up times (including the use of repeated measures regression models), (b) repeating the analysis for the primary outcome adjusting for any observed imbalance between arms of potential predictor variables at the level of the individual and/or the UBS (introduction of the latter would involve the use of suitable multi-level models) and (c) similar analyses for secondary outcomes for participants at 8 and 12 months. In addition to the adjustment for clustering by (allocated) UBS in the primary analyses, multi-level models will be employed to investigate the extent to which the primary analyses were affected by various clustering effects—UBSs, FHTs and CHWs.

#### Economic analysis

The primary economic analysis will take a Brazilian public health system perspective. Quality-adjusted life years (QALYs) over the 12-month follow-up period will be calculated based on EQ-5D-5L responses using the most appropriate EQ-5D-5L value set available at the time of the analysis. In the primary analysis, the incremental costs and QALYs of the psychosocial intervention will be combined to estimate the incremental net monetary benefit of the intervention at a range of ‘willingness-to-pay’ thresholds. We will also estimate cost-effectiveness acceptability curves to illustrate the probability that the intervention is cost-effective at a range of thresholds. In a secondary analysis, we will estimate the incremental cost-effectiveness ratio of the psychosocial intervention compared to enhanced usual care using the primary clinical outcome measure (i.e. cost per patient recovered at 8 months).

### Interim analyses {21b}

In terms of the statistical and economic analyses of the trial, we do not envisage conducting any between-arm comparisons as interim analyses. The database will be locked by the transaction manager after quality controls. The only reason to check this database before completing the trial is when there is a major concern about the health and/or wellbeing of a participant. However, this would involve only the participant concerned, and this information may need to be shared with others involved in the safety of participants.

### Methods for additional analyses (e.g. subgroup analyses) {20b}

Exploratory subgroup analyses will be undertaken to investigate potential differential effects of the intervention according to pre-specified characteristics of the participants. While the list of potential effect modifiers will be finalised prior to publishing the Statistical Analysis Plan for the trial, the current plan is to consider at least some of the following: gender, age (as a continuous or grouped variable), educational level, presence of co-morbid physical illnesses and severity of depression (the continuous PHQ-9) at baseline. Such analyses will be interpreted cautiously, partly because we do not have an overwhelming single prior hypothesis for these analyses and partly because statistical power will be limited for the required interaction effects within the relevant regression models.

### Methods in analysis to handle protocol non-adherence and any statistical methods to handle missing data {20c}

Sensitivity analysis using multiple imputation models for missing values will be conducted to investigate the potential effects of missing data and attrition. Further secondary analyses will compare individuals according to the intervention they received, accounting for any selection effects after random allocation using complier-average causal effect methods employing instrumental variables regression models. These analyses will first use a binary definition of an ‘adequate dose’ of the intervention, as follows: in the low-intensity group, a minimum of 6 sessions; in the high-intensity group, a minimum of 7 sessions. We will also conduct these analyses according to the number of sessions attended.

### Plans to give access to the full protocol, participant level-data and statistical code {31c}

Summary information of the datasets will be published in relevant research data repositories and directories. Our data will be locked until our main hypothesis-based analyses are completed and published or for 3 years after completion of data collection (whichever is earlier), after which the data will be accessible for external use. The research team has interest in mutually sharing and pooling metadata with other research groups. Data will not be shared with any other source or used for any other purpose other than those approved by the Ethics Committees.

We plan to maximise the availability of research data with as few restrictions as possible, ensure that data should be released in a format that conforms to agreed standards, especially where this allows interoperability with other relevant datasets, require that all users of data acknowledge the sources of their data and conform with the terms and conditions under which they accessed the original data, and put in place any required limitations on data sharing that serve to safeguard the privacy of research participants.

We will implement a data management and sharing plan. This plan will specify the roles, responsibilities and relationships of key persons involved in the research: the applicant, sponsors and collaborators, research team members, research participants and research institutions.

The joint principal investigators, in consultation with a Data Access Committee, will be responsible to grant access to the data, which will be in compliance with sponsors’ regulations. There will be a form to fill to request access to the data. The joint principal investigators will review and approve any such requests. Thus, study data will be made available to researchers after written requests spelling out the specific aims and plan of analysis. If there are conflicts of interest or requests to use data for purposes other than those approved by the Ethics Committee, we will seek advice from them to resolve any such disputes if they ever occur. Data sharing will be in compliance with the principles outlined in Good Practice Principles for Sharing Individual Participant Data from Publicly Funded Clinical Trials. External users will be bound by appropriate data sharing agreements with the Ethical Committee and in line with sponsors and funders’ policies.

## Oversight and monitoring

### Composition of the coordinating centre and Trial Steering Committee {5d}

#### Coordinating centre

University of São Paulo, São Paulo, Brazil.

#### Trial Steering Committee (TSC)

The TSC will meet once a year and is composed of:

##### Independent chair

Professor Simon Gilbody, Professor of Psychological Medicine and Health Services Research, University of York, UK.

##### Members

Dr. Marcia Scazufca, Study PI—FAPESP-Brazil, USP Medical School, University of São Paulo, Brazil.

Professor Ricardo Araya, Study PI—MRC-UK, Institute of Psychiatry, Psychology and Neuroscience, King’s College London, UK.

Professor Tim Peters, Study Co-investigator, Bristol Medical School, University of Bristol, UK.

Professor Roberto Lourenço, Director of the Department of Geriatrics, Professor of Geriatrics, Pontifical Catholic University of Rio de Janeiro, Brazil (Independent member).

Rodrigo Fonseca Martins Leite, M.D., Lecturer, Municipal University of São Caetano do Sul, Brazil (Independent member).

Walter Freitas Junior, M.D., Director of the Division of Health Education Management and Coordinator of the Multi-professional Residency Committee—Escola SUS, Guarulhos Health Secretariat, Brazil (Independent member).

Paula Verônica Martini Maciel, D.D.D., Coordinator of the Medical Residency Committee and Member of the Technical Staff of Escola SUS, Guarulhos Secretary of Health, Brazil (Independent member).

### Composition of the Data Monitoring Committee, its role and reporting structure {21a}

#### Data Monitoring Committee (DMC)

##### Independent chair

Dr. Claudia de Souza Lopes, Associate Professor, Institute of Social Medicine, Rio de Janeiro State University, Brazil

### Adverse event reporting and harms {22}

In this study, serious adverse event (SAE) data will be collected—that is, any untoward medical occurrence that is believed by the investigators to be causally related to study intervention. SAEs can result in any of the following: death, attempted suicide, life-threatening situations (that is, immediate risk of death) or significant physical or emotional disability/incapacity. SAEs of individuals who discontinued participation will not be reported. CHWs and sub-contracted RAs will be trained to obtain SAE information from visits. The intervention coordinator will be responsible for the completion of SAE forms and contacting the principal investigator, when necessary. Where appropriate, reports should be forwarded promptly to the Ethics Committee of the USP Medical School, with a copy to the DMC chair. A SOP for SAE recording, management and reporting was also developed to guide the team.

We do not think that this trial poses any considerable risks to trial participants. The intervention to be evaluated adds to existing usual care, and the trial does not prevent family doctors or other FHT professionals initiating or changing treatment for depression or any other health problem. None of the components to be tested is considered harmful.

The risk of suicide among depressed participants will be assessed regularly, and participants at high risk at any stage will receive immediate support from the sub-contracted RA or CHW, something that does not occur at present. CHWs and sub-contracted RAs will be trained in assessing risk with a standardised protocol, and CHWs will also receive ongoing supervision of their cases. A safety plan has been developed to deal with these emergencies and tested during our pilot study with two participants at risk of suicide while receiving the pilot psychosocial intervention. The safety plan was applied successfully by the CHWs in the pilot study. As part of this safety plan, these participants were seen by the family doctor for further assessment and subsequently managed by the FHT.

### Frequency and plans for auditing trial conduct {23}

We will audit the recruitment (and retention) rates across arms to investigate the extent of any differential recruitment/retention and if any bias might accrue in terms of depression scores. The DMC will be informed of the findings of these checks.

### Plans for communicating important protocol amendments to relevant parties (e.g. trial participants, ethical committees) {25}

Any modifications on the study design, recruitment, study procedures, intervention, data collection and data analysis agreed between the research team, TSC and DMC will be communicated to the Ethical Committees and funders. Guarulhos health system managers will also be notified and need to agree with any modification related to the daily functioning of their health units. Protocol amendments will only be implemented after appropriate approval has been received.

## Dissemination plans {31a}

We expect to publish the results in internationally competitive journals, something in which we have a good track record [18, 19, 21, 55–57]. We will present the project and results at local, regional and international conferences in any of the relevant disciplinary fields. Additional dissemination will be through the Centre for Global Mental Health websites and programme of extramural activities. We will engage King’s College London’s press office and public engagement coordinator. There will be media engagements and other dissemination activities. Dissemination in Brazil will be assisted by the press officers of the institutions involved. The core group of investigators will convene 1 month before the end of the study to discuss results and plans. In parallel, we will organise dissemination meetings and workshops with main stakeholders to discuss results and potential impact of scaling up. We will arrange meetings of our experts with our main stakeholders to assist us in the process of enlisting their support and persuading them of the importance of this and other similar future projects. We have an intensive plan of dissemination of the results involving policy-makers as well as potential funders/donors in this field.

## Discussion

The global burden of depression is rising, and the elderly population presents an increased risk for experiencing this disease. In many LMIC, the health systems are unable to meet the mental health demand, impacting negatively on the identification and treatment of this population [58]. There is an urgent need for feasible and affordable treatment programmes based in primary care for later life depression.

A two-arm cluster, non-randomised controlled trial pilot study [22] was conducted in two UBSs in São Paulo to certify the feasibility of recruitment, assessments, randomisation and acceptability of the proposed psychosocial intervention. Twenty-five and 33 participants were recruited for control and intervention arms, respectively, and the 17-week psychosocial intervention was delivered by CHWs and nurse assistants. The follow-up rates were higher than 92% and both arms showed a lower PHQ-9 score mean around 30 weeks after inclusion, but there was a substantially improvement in depression symptoms in the intervention UBS. Qualitative research also indicated that the intervention was acceptable to both participants and health professionals and the participants enjoyed especially being seen at home regularly, the activities and structure of the intervention, and the use of technology and multimedia resources.

As far as we are aware of, this will become the first large pragmatic trial to evaluate an intervention to improve the management of depression among older adults delivered by existing non-medical personnel in LMIC/Latin America. As such, it will provide unique and important information to Brazilian as well as other policy-makers from countries experiencing similar problems. Potentially, this intervention could alleviate the suffering of millions of people who currently receive little or no help for their mental health problems.

## Trial status

Protocol version 2.0 from 16 March 2020. The recruitment of the first wave started on 23 May 2019 and of the second wave on 20 January 2020. Since 12 March 2020, the recruitment of new participants is paused and the psychosocial intervention conducted by the CHWs has been stopped due to the emergence of the COVID-19 pandemic. The follow-up assessments are being conducted by phone. We have plans to restart the second wave when it is safe for everyone involved. If the COVID-19 pandemic does not subside and therefore does not allow the continuation of the RCT as described in this protocol, we will discuss amendments and follow appropriate approval from the relevant parties.

## Data Availability

The data will be locked until the end of the data collection, and there will be only one main analysis at the end of the study. We may also analyse the baseline data to provide information that the main stakeholders are interested in as a part of our strategy to begin sensitising policy-makers for future scaling up of the programme.

## Abbreviations

AUDIT-C: Alcohol Use Disorders Identification Test-Consumption
BADS-SF: Behavioural Activation for Depression Scale—Short Form
CHW: Community health worker
DMC: Data Monitoring Committee
EQ-5D-5L: European Quality of Life five-dimensional questionnaire, five-level version
FHS: Family Health Strategy
FHT: Family Health Team
GAD-7: General Anxiety Disorder-7
ICECAP-O: ICEpop CAPability measure for Older people
LMIC: Low- and middle-income countries
LSNS-6: Lubben Social Network Scale-6
PHQ-9: 9-item Patient Health Questionnaire
QALYs: Quality-adjusted life years
RA: Research assistant
SAE: Serious adverse event
SOP: Standard operating procedure
TSC: Trial Steering Committee
UBS: Basic Health Unit (Unidade Básica de Saúde)
3-item UCLA: 3-item University of California, Los Angeles loneliness scale
