# Correction to: A collaborative care psychosocial intervention to improve late life depression in socioeconomically deprived areas of Guarulhos, Brazil: the PROACTIVE cluster randomised controlled trial protocol

**DOI:** 10.1186/s13063-020-04957-0

**Published:** 2020-12-16

**Authors:** Marcia Scazufca, Carina Akemi Nakamura, Tim J. Peters, Maiara Garcia Henrique, Antônio Seabra, Ehidee Gomez La Rotta, Renato M. Franzin, Daniele Ferreira Martins, Pepijn Van de Ven, William Hollingworth, Ricardo Araya

**Affiliations:** 1 Hospital das Clinicas HCFMUSP, Faculdade de Medicina, Universidade de Sao Paulo, Sao Paulo, Brazil; 2 Faculdade de Medicina FMUSP, Universidade de Sao Paulo, Sao Paulo, Brazil; 3 University of Bristol Medical School, Bristol, England; 4 Departamento de Engenharia Eletrica, Escola Politecnica, Universidade de Sao Paulo, Sao Paulo, Brazil; 5 Health Research Institute, University of Limerick, Limerick, Ireland; 6 Institute of Psychiatry, Psychology and Neurosciences, King’s College London, London, England

**Correction to: Trials 21, 914 (2020)**

**http://orcid.org/10.1186/s13063-020-04826-w**

Following publication of the original article [1], we were notified that the academic title for two of the authors, as well as the affiliation of Pepijn Van den Ven need to be corrected.

- Originally published academic titles:

Paula Verônica Martini Maciel, D.D.D.

Walter Freitas Junior, M.D.

- Correct academic titles:

Paula Verônica Martini Maciel, M.D.

Walter Freitas Junior, D.D.D.

- Original affiliation:

Faculty of Science and Engeneering, University of Limerick, Limerick, England

- Corrected affiliation:

Health Research Institute, University of Limerick, Limerick, Ireland

The original article has been corrected.
